# Prediction of Tensile Strength in Friction Welding Joins Made of SA213 Tube to SA387 Tube Plate through Optimization Techniques

**DOI:** 10.3390/ma12244079

**Published:** 2019-12-06

**Authors:** Senthil Kumaran S, Kathiravan Srinivasan, Srinivasan Narayanan, Alex Noel Joseph Raj

**Affiliations:** 1 Department of Manufacturing Engineering, School of Mechanical Engineering, Vellore Institute of Technology, Vellore 632014, India; senthilkumaran.s@vit.ac.in (S.K.S.); srinivasan.narayanan@vit.ac.in (S.N.); 2 School of Information Technology and Engineering, Vellore Institute of Technology (VIT), Vellore 632014, India; kathiravan.srinivasan@vit.ac.in; 3 Key Laboratory of Digital Signal and Image Processing of Guangdong Province, Department of Electronic Engineering, College of Engineering, Shantou University, Shantou 515063, China

**Keywords:** FWTPET, ANOVA, design of experiments, friction welding, genetic algorithm, advanced welding, joining processes

## Abstract

In modern times, the Industry X.0 has emerged as the paradigm that has become the core of digital technology-driven business organizations. Further, this paper establishes a tube to tube plate friction welding technology with the help of deploying an external tool, also known referred to as the FWTPET scheme. Besides, the SA213 tube and SA387 tube plate were combined by employing a unique interference fit technique. Also, the strength of this combined portion was assessed with and without the aid of a holding block. Subsequently, the analytic optimization approaches like genetic algorithm, analysis of variance, and Taguchi L_9_ orthogonal array design were deployed in the prediction of the optimum joining strength. Moreover, the input parameters include the projection of the tube (mm), the rotational speed of the tool (rpm), and depth of cut (mm); besides, the tensile strength is considered as the output parameter. Also, the grain size distribution around the weld zone and the presence of base metal were measured through an optical microscope as per ASTM linear intercept method. Further, it is evident that grain refinement had occurred in the weld zone, which in turn increases the tensile strength. The exceptional weld strength (tensile strength) was obtained when joining of SA213 tube and SA387 tube plate through interference fit using a holding block without a hole in the tube. Experimentally, it was found that the achieved tensile strengths were 836.8 MPa (without a hole) and 789.35 MPa (with hole) using the holding block, respectively. Additionally, it was found that in the absence of a holding block, the achieved tensile strength is 762.2 MPa (without a hole), and 700.8 MPa (with a hole), correspondingly. The deviation of tensile strength between the predicted (genetic algorithm) and experimental was found minimal. Therefore, for achieving this strength, the suitable operating parameters set include the rotational speed of the tool (1300 rpm), projection of the tube (1 mm), and depth of cut (0.5 mm) with backing block configuration.

## 1. Introduction

In recent years, friction welding (FW) technology has become widely popular for several industrial and manufacturing applications, including construction, electronics, aerospace, power plants, and automobiles. Besides, it is a prominent tool for any application that involves combining metals by utilizing solid-state welding. Further, this approach is highly deployed in production units, since it has the capability of combining similar and dissimilar things with superior joint integrity. The several industrial components such as pressure vessels, space crafts, earthmovers make use of conventional welding techniques. The main feature of FW is that excessive heat generation leads to a softening state that interacts and produces a good quality weld [1,2,3]. In general, to achieve quality joint strength, an appropriate combination of the input process parameters is needed. Fusion welding makes use of heat to join the materials [4,5]. The current literature suggests the fact that friction welding offers a potential advantage than fusion welding [6,7]. The tube to tube plate friction welding technology is deployed by utilizing an external tool that is abbreviated as the FWTPET scheme and patent was granted in the year 2008 [8].

Several authors, including some of the members from this work, have carried out extensive research and for a detailed review, refer to reference [5]. The technique of the FWTPET scheme had been remarkably proven, and it offers numerous benefits than conventional friction welding technology [9]. The absence of solidification cracks and porous structure makes the FWTPET scheme much more attractive than the conventional friction welding approach. Moreover, welding process parameters’ and its statistical significance was established using a combination of L27 orthogonal array of Taguchi method, neural model, and genetic algorithm with and without supporting block has been explored [10,11]. Moreover, the work in [7] accomplishes the finite-element determining the deformation behavior, stress, and strain distribution with the aid of the ABAQUSTM software [7]. There are several current progressive works in the area of metallic materials processes of welding and joining [8,9,10,11,12,13,14,15,16]. 

Primarily, this research work establishes the FWTPET scheme for combining the SA213 (T12 grade tube) and SA387 (T22 grade tube plate) by deploying the approach referred to as interference fit. The novelty of this work is the application of the FWTPET technique for combining the SA213 tube and SA387 tube plate using the interference fit technique and its analytical validation. So far, one of the present authors in this research group had already published specific works in FWTPET with different variations such as backing block, types of fit, and with the hole (WH)/without a hole (WOH). Moreover, the vast body of published articles involving both experimental and analytical methods have dealt with clearance fits [9,12,13,14] with a backing block with a tube-holes arrangement. Till now, very few works were reported using the interference fit method [8,10,15]. Herein, a comparison of all the variants of FWTPET was studied with an experimental and analytical approach. Besides, the constant quest for achieving higher tensile strength resulted in adopting an interference fit method, deliberated in the experimental section. Additionally, in this research work, the novel FWTPET scheme for combining the SA213 tube and SA387 tube plate by deploying the interference fit method for the box type heat exchanger application was attempted. Besides, the tensile strength value was predicted numerically using ANOVA and Genetic Algorithm techniques.

## 2. Experimental Setup

This research work primarily concentrates on joining the metal SA213 (T12 grade tube) and SA387 (T22 grade tube plate). Table 1 and Table 2 signify the alloy chemistry of the SA 213 tube and SA 387 tube plate. Further, it has to be noted that the FWTPET schemes are developed in-house and consist of tools configuration, a fixture with the help of a modified milling machine. Herein, the projection of the tube becomes a vital factor that conditions the performance of successful welding in tube to tube plate. We introduce an exterior tool (tungsten carbide) for welding the tube to tube plate assembly, and Table 3 portrays its alloy chemistry of tungsten carbide.

**Figure 1:**
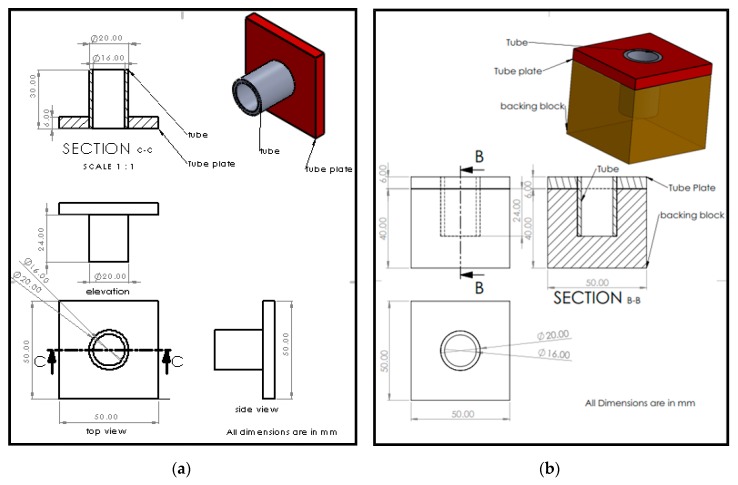
(**a**) Diagram of the tube to tube plate setup arrangement; (**b**) diagram of the backing block (supporting block).

The exterior tool is made up of tool-pin and shoulder [9]. It is assembled in a modified milling machine tool holder arrangement. Figure 1a,b depict the diagram of the tube to tube plate arrangement and backing block along with dimensions.

**Figure 2:**
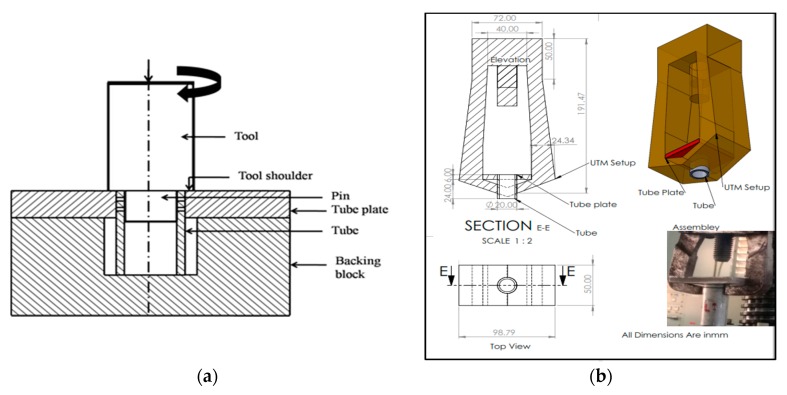
(**a**) Schematic diagram of the Interference fit method; (**b**) diagram of tensile test fixture along with tested specimen.

An interference fit (press fit or friction fit) is used to join two similar/dissimilar parts by the friction welding process. This technique is commonly applied for joining shaft and hole assembly. An interference fit creates a high temperature than the clearance fit in the FWTPET process. The FWTPET process was carried out by an interference fit. The process was explained in the schematic diagram given below (Figure 2a). The basic principle behind joining the tube to tube plate, when the tool is rotated with suitable input rotational speed as per the welding condition, and tool shoulder once touches the tube plate, when it moves towards the downward direction. When contacting the tool shoulder on the tube plate, enormous amount of heat gets generated due to friction. Therefore, the flexible flow of the metal takes place in the direction towards the center of the tool axis. The circular shaped pin restricts the metal flow and acts as an anvil for the surrounding portions of the workpiece. It creates suitable bonding and pressure at the weld interface, and the tool is retracted after a pre-determined time. A tensile test is probably the most fundamental type of mechanical test which can perform on welding samples, and the assembly of tensile strength sample is shown in Figure 2b. A modified UTM was used to take a tensile strength test for nine samples. A rod and hook-like strength were the modification of the UTM developed in-house. By pulling the workpiece, it is easy to determine how the workpiece will react to a tensile force.

**Figure 3:**
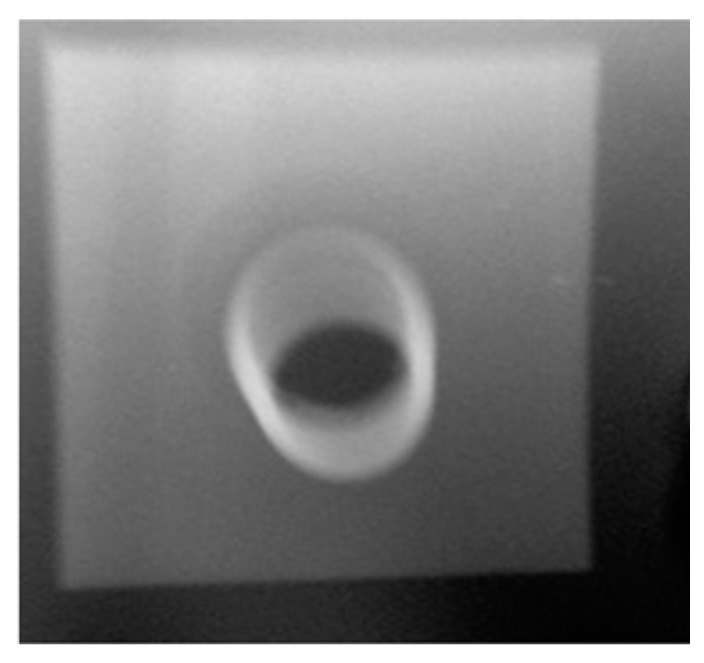
Radiography test sample FWTPET sample of rotational tool speed (1300 rpm), projection (1 mm), and depth of cut (0.5 mm).

The rough and fine scratch of the microstructure samples is removed by using dry belt polisher and different grade emery sheet. Finally, the surfaces were polished using alumina powder and fine diamond paste. For the microstructural investigation, the Nital solution was chosen, and microstructure was developed. Because of solid-state welding, the metal mixed, and it resembles by air quenching process with no cracks and porosity shown in Figure 3.

## 3. Approaches for Optimization

L_9_ orthogonal array was analyzed with these nine samples at different weld conditions. Table 4 represents three factors and levels of Taguchi L_9_ orthogonal array. Table 5 shows that the experimental layout contains nine experimental runs at different input parameters. For ensuring statistical repeatability for each condition, minimum 3 specimens were tested. Due to brevity, few results were presented. The genetic algorithm was the short outcomes of newton type optimizers. This technique was a non-traditional technique that predicts the values from the iteration method. The function value and the derivatives concerning the parameters optimized are used to take a step in a suitable direction towards a local maximum or minimum. It generates a population of points at each iteration, and the best point in the population approaches an optimum solution.

## 4. Results and Discussions

In this research work, the investigation of FWTPET was carried out by an interference fit method. Further, the results were compared with and without the presence of a supporting block and with and WOH in SA213tube.

### 4.1. Tube WOH by Employing Backing Block Arrangement

L_9_ orthogonal array process parameters reveal in Table 6.

#### 4.1.1. Signal to Noise Ratio

**Figure 4:**
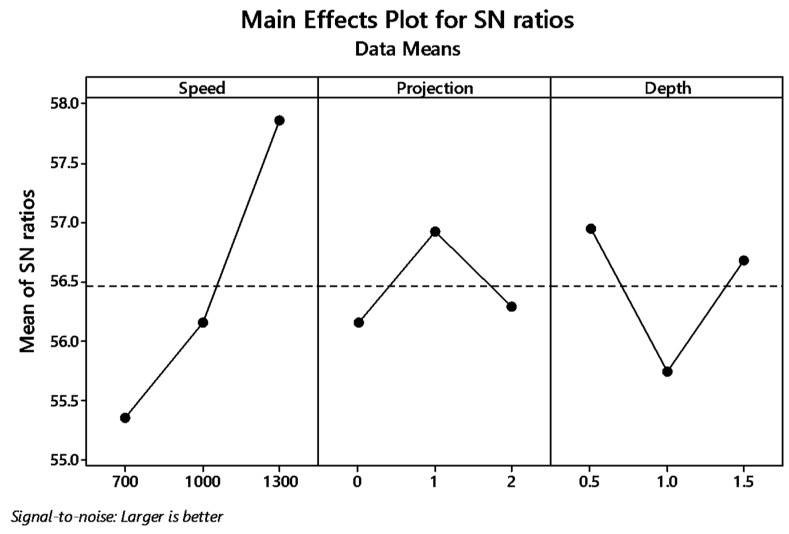
Main effects plot for S/N ratio of (without a hole (WOH)) on the tube using backing block.

**Figure 5:**
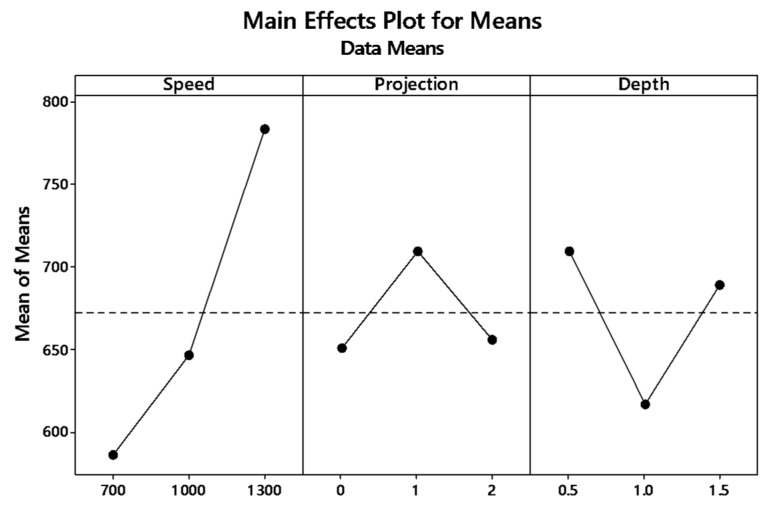
Main effects plot for means of (WOH) on the tube using backing block.

The graph plot of signal to noise ratio and means is illustrated in Figure 4 and Figure 5. The desired signal to the background noise was examined by the Larger is better criteria. Further, this is because when the tensile strength needs to be larger so that the welding samples had better-joining strength. MINITAB is the statistical software that is used to examine the most significant parameters and contributions of each parameter. 

In the graph, the optimum joint strength input parameters were plotted. The most significant parameter 1300 rpm tool rotation speed, 1 mm tube projection, and 0.5 depth of cut produces maximum tensile strength of 2355 MPa. By ranking process, depth of cut is the significant parameter of better weldability shown in Table 7.

Both means and signal to noise ratio graph, the parameters such as speed, projection, and depth of cut produce the maximum output tensile strength.

#### 4.1.2. Analysis of Variance (ANOVA) Approach

**Figure 6:**
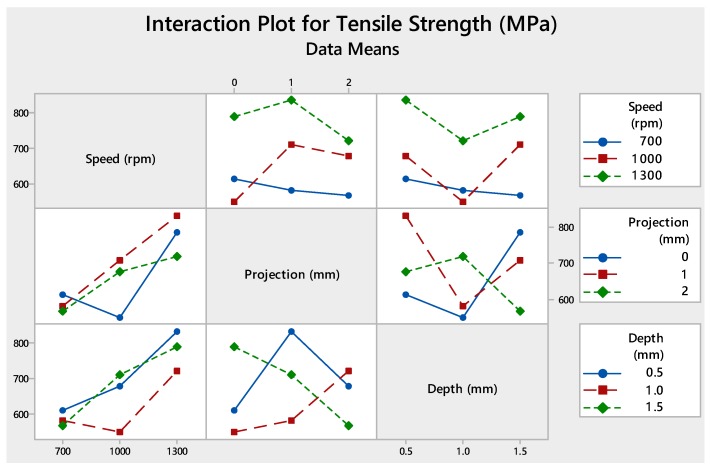
Interaction plot of without-hole on the tube using backing block.

**Figure 7:**
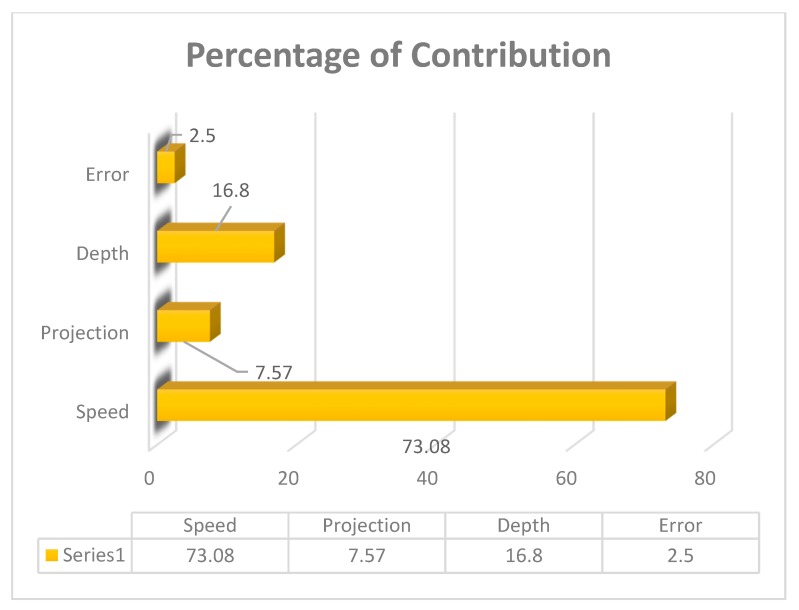
Percentage of the contribution of (WOH) on the tube using backing block.

Analysis of variance was being performed to find out the contribution of each parameter by using the software MINITAB (MINITAB 18, Pennsylvania, USA). Table 8 shows the percentage contribution of process parameters. Moreover, Figure 6 and Figure 7 show the interaction plot and graphical representation of process percentage. As per the result, the maximum contribution among the input parameters was tool rotational speed (73.08%) followed by the depth of cut (16.80%) and tube projection (7.57%).

#### 4.1.3. Genetic Algorithm Approach

**Figure 8:**
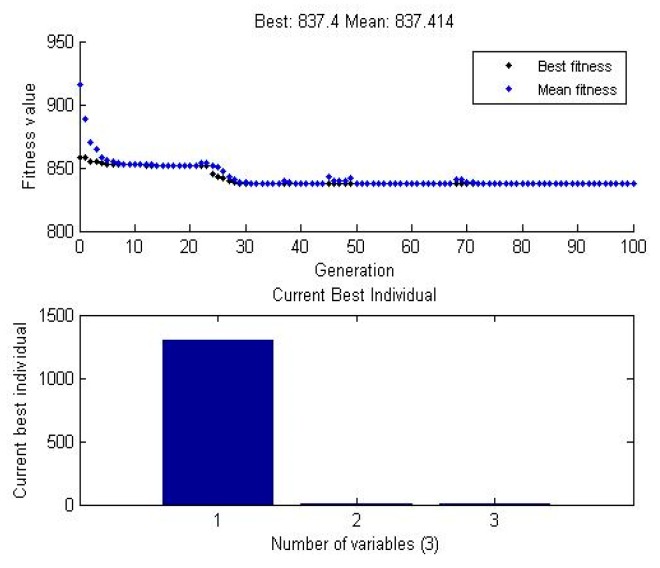
GA graph plot for data means (WOH) in the tube using backing block.

In our study, the essential elements of GA consist of a value of Tool rotational speed, Projection, depth of cut. In this study, the best value predicted by GA was 837 MPa, and the means value was 837.414 MPa for the best individual 1300 rpm tool rotational speed, 1 mm tube projection, and 0.5 mm depth of cut. The predicted value was shown in Figure 8. The comparison values of GA and experimental values were tabulated in Table 9. The parameters used in GA are; population size = 100, length of chromosome = 20, selection operator = stochastic uniform, crossover probability = 0.8, mutation probability = 0.2, and fitness parameter = tensile strength. The objective function is given by Tensile strength = f (Speed, Projection, Depth).

### 4.2. Tube with Hole (WH) by Employing Backing Block Arrangement

#### 4.2.1. Signal to Noise Ratio

In this present investigation, the tube was prepared WHs on the circumference of the tube. The tube and tube plate was assembled tightly. Previously, the tube and tube plate was cleaned manually. In this investigation, the maximum tensile strength was obtained as 789.35 MPa, and its input parameter was 1300 rpm, 1 mm projection, and 0.5 mm depth of cut. The input and output parameters were shown in Table 10, given below. Data mean values and signal-to-noise (S/N) ratio values for input parameters have been calculated to access the factor in response. 

**Figure 9:**
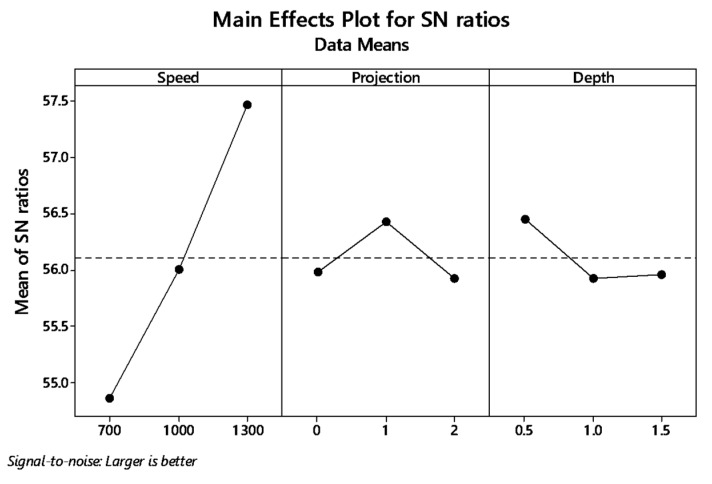
Main effect plot for S/N ratio (WH) on the tube using backing block.

**Figure 10:**
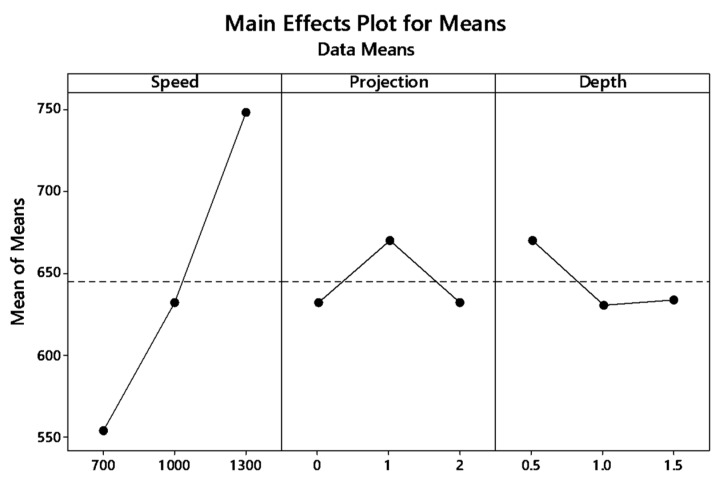
Main effect plots for means of with-hole on the tube using backing block.

From the signal to noise ratio, the criteria “Larger is better” was selected because the maximum tensile strength was to be calculated. Table 11 provide the signal to noise ratio to qualify the values corresponds to better quality features. The graph plot for S/N ratio and data mean was picturized in Figure 9 and Figure 10, respectively.

#### 4.2.2. Analysis of Variance

**Figure 11:**
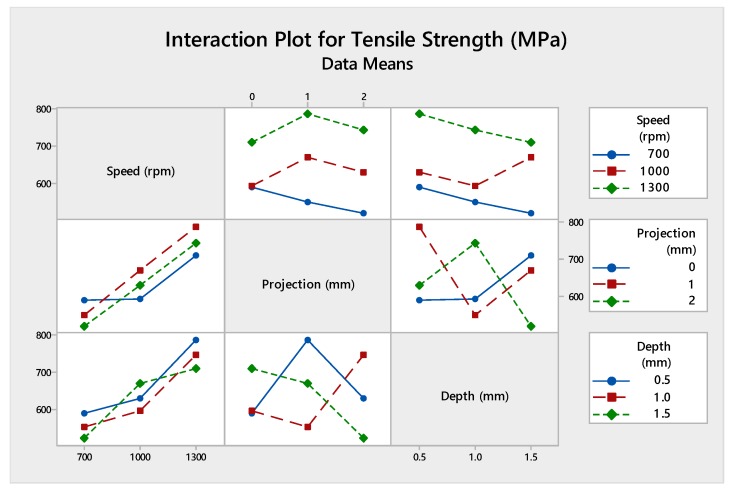
Interaction plot of (WH) on the tube using backing block.

**Figure 12:**
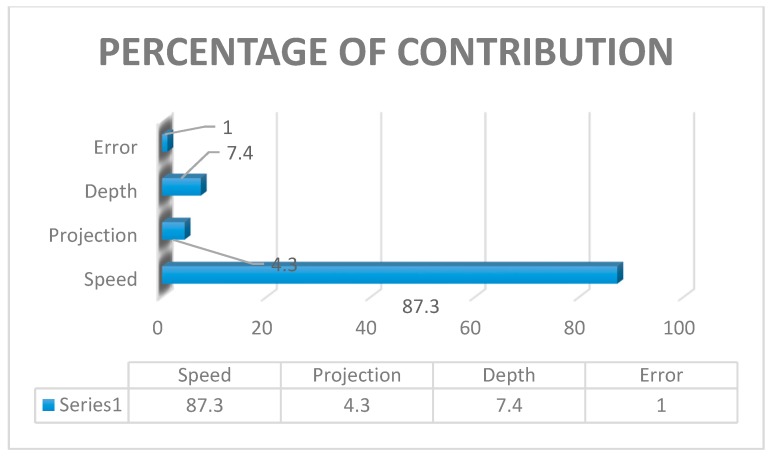
Percentage of Contribution of (WH) on the tube using backing block.

The reason for identifying the analysis of variance in statistical method to explore the contribution of input parameters utilizing the percentage shown in Table 12. In this present work, the maximum contribution was tool rotational speed (87.3%). Further, this was followed by the plunge depth (7.40%) and tube projection (4.30%). The graph plot of the interaction of process parameters was shown in Figure 11. The pictorial representation of the inheritance percentage was shown in Figure 12.

#### 4.2.3. Genetic Algorithm Approach

**Figure 13:**
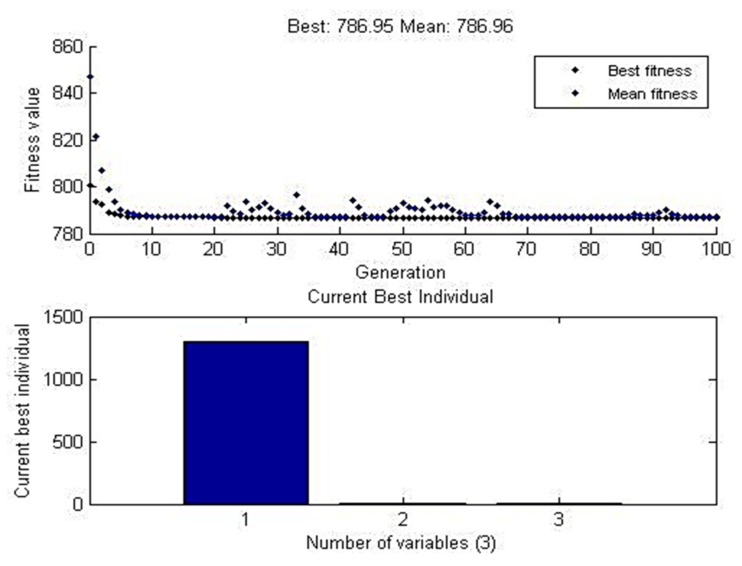
GA graph plot for data means of (WH) on the tube using backing block.

The genetic algorithm was a non-traditional technique based on the principle of natural genetics. It was effortless and eased in operation. In this study, Tensile strength was predicted by GA as 786.95 MPa and the mean value corrected to 786.96 MPa. The comparison values were iterated with the experimental value, and it was tabulated in Table 13. The graph plot was shown in Figure 13.

### 4.3. Tube (WOH) in the Absence of Backing Block

In this present investigation, the tube and tube plate was arranged perpendicularly, and the FWTPET process was carried out without employing the supporting block. The tube has no holes on the circumference. The experiment was conducted by Taguchi L_9_ orthogonal array. 

#### 4.3.1. Signal to Noise Ratio

The tensile strength was calculated for nine samples by UTM. The maximum tensile strength was obtained as 762.2 MPa by the input parameters such as 1300 rpm tool rotational speed, 1 mm projection, and 0.5 mm depth of cut. The output parameter tensile strength was tabulated in Table 14, given below.

**Figure 14:**
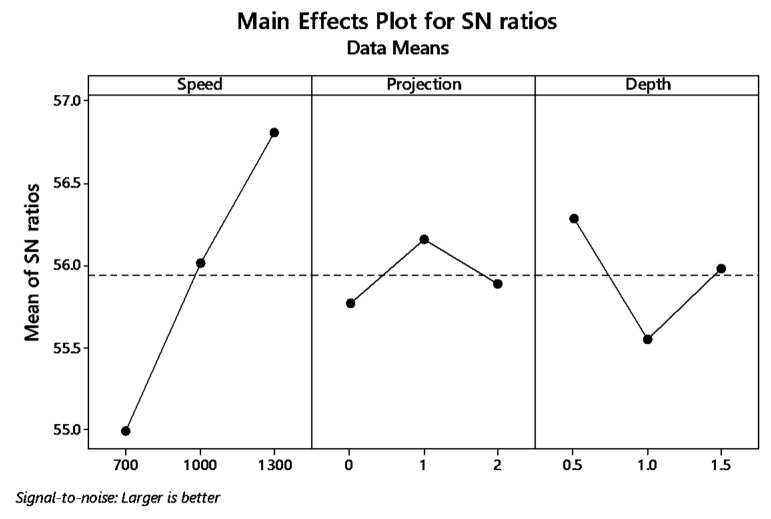
Main effects plot for S/N ratio of without-hole on the tube in-absence of the backing block.

**Figure 15:**
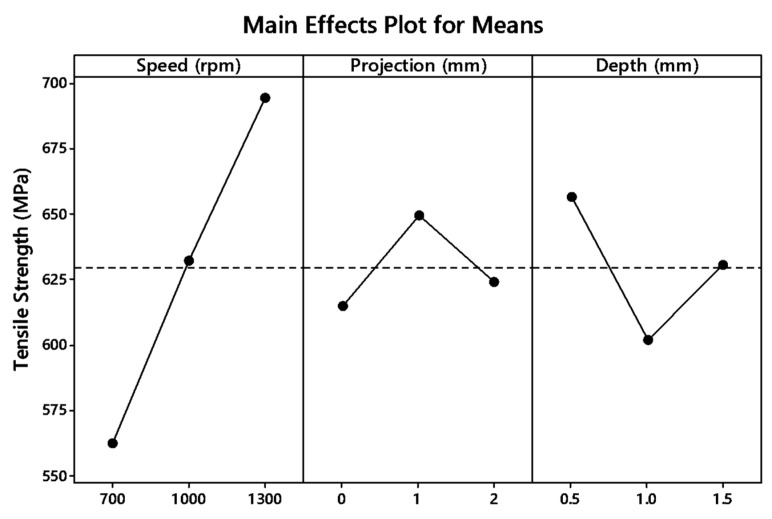
Main effects plot for means of without-hole on the tube in-absence of the backing block.

Figure 14 and Figure 15 show the main effect plot for S/N ratio and means for WOHs on the tube in the absence of a backing block. By the graph plot, once again shows the significant input parameters for producing tensile strength. The signal to noise ratio ranking process are tabulated in Table 15 shows that speed was the rank one input parameter.

#### 4.3.2. Analysis of Variance (ANOVA) Approach

The contribution of each process parameters would be found out by using the ANOVA technique (MINITAB 18, Harrisburg, PA, USA). 

**Figure 16:**
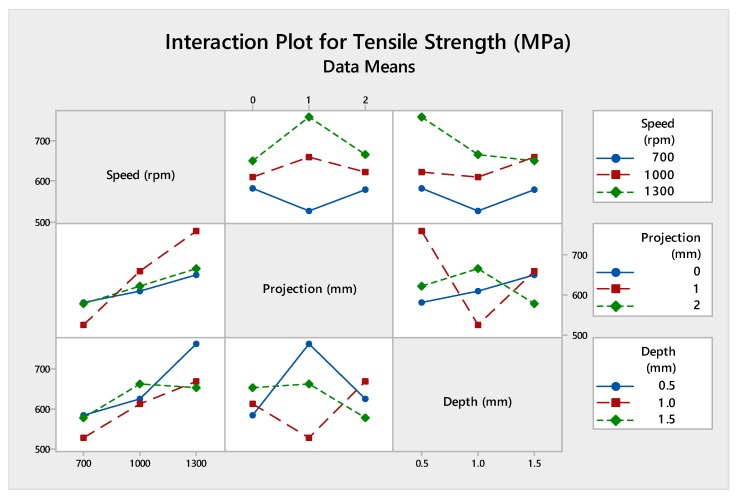
Interaction plot of (WOH) on the tube in-absence of the backing block.

**Figure 17:**
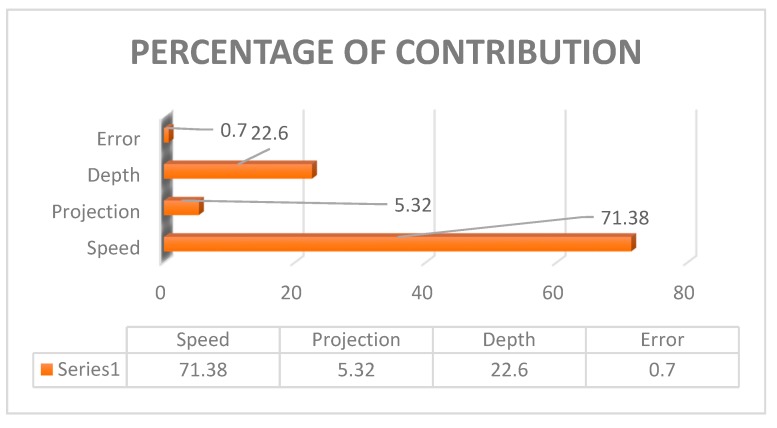
Percentage of contribution of (WOH) on the tube in-absence of the backing block.

Figure 16 shows the interaction plot contains input parameters of speed, projection, and depth of cut which outgrowth the optimal tensile strength values. Also, this put the error into zero, produces an absolute value of the tensile strength. Table 16 shows the analysis of variance contribution percentage by using each parameter adjacent SS values and total adjacent SS values. Figure 17 was the graphical representation of the contribution percentage.

#### 4.3.3. Genetic Algorithm Approach

**Figure 18:**
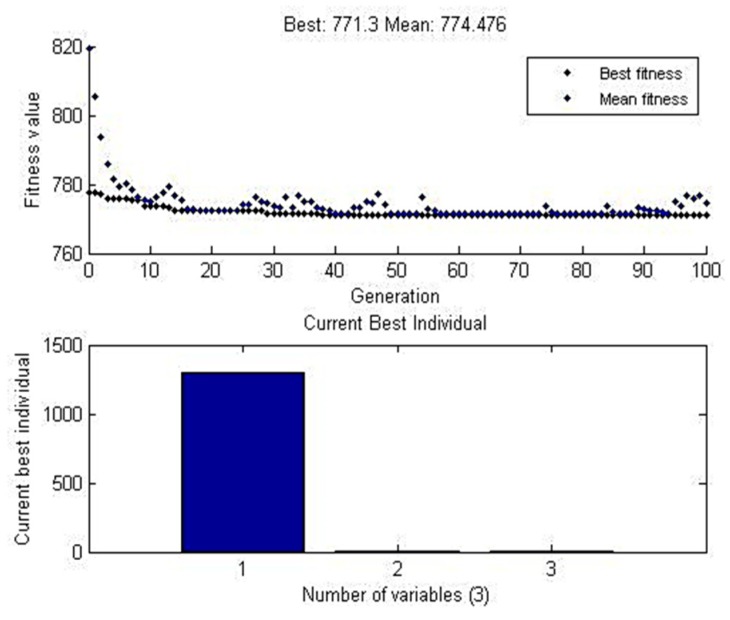
GA graph plot for data mean (WOH) on the tube in the absence of backing block.

GA works under the principles of natural genetics that state the concept, “Fit parents would yield fit offspring”. The maximum function was found based on the variation of X_1_-n beginning with one or more starting points. In this present study, the maximum output values were predicted as 771.3 MPa, and its data mean was 774.476 MPa shown in Figure 18. The comparison table between the experimental values and software predicted values were shown in Table 17.

### 4.4. Tube (WOH) in the Absence of the Backing Block

In this present study, the tube and tube plate was welded by the FWTPET process. The tube was prepared by holes in its circumference. The experiment was conducted by L_9_ orthogonal array. The input and output parameters of the FWTPET process was tabulated in Table 18.

#### 4.4.1. Signal to Noise Ratio

**Figure 19:**
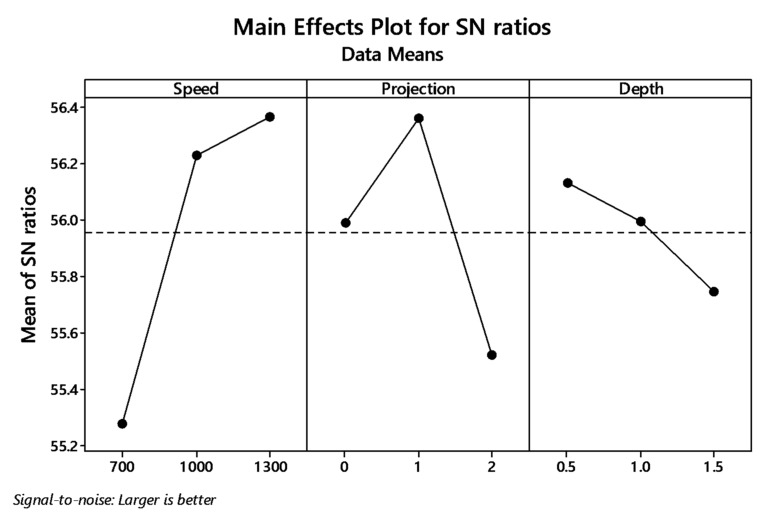
Main effect plot for S/N ratio with-hole on the tube in the absence of the backing block.

**Figure 20:**
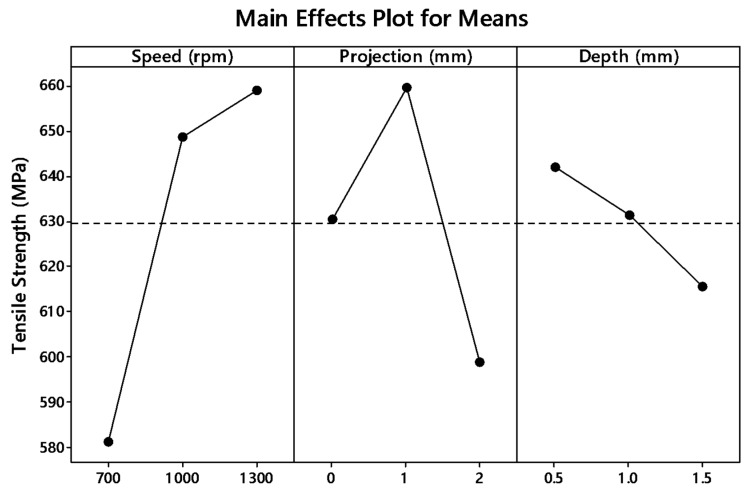
Main effect plots for means with-hole on the tube in the absence of the backing block.

The tensile strength was identified for all nine welded samples. From the table, the maximum tensile strength was obtained as 700.8 MPa. The input parameters were 1300 rpm tool rotational speed, 1 mm projection, and 0.5 mm depth of cut to find out optimum joint strength. The SN ratio and data mean were calculated, and a separate graph was plotted in Figure 19 and Figure 20 which indicates the joint strength was at its maximum, with tool rotational speed of 1300 rpm, tube projection of 1 mm, and plunge depth of 0.5 mm. Table 19 provides the response table for the SN ratio that contains more significant value denotes the better criteria.

The welded samples achieve maximum tensile strength mainly by the input parameter tool rotational speed (1300 rpm). The friction creates when the tool shoulder touches the tube plate. The heat dissipates efficiently from the tube to the tube plate. The molten metal flows towards the axis, and it fills the holes prepared on the circumference of the tube.

#### 4.4.2. Analysis of Variance Approach

**Figure 21:**
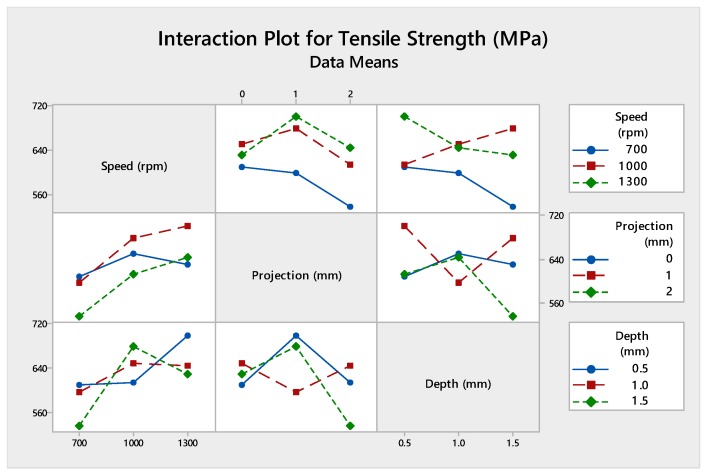
Interaction plot with-hole on the tube in the absence of the backing block.

**Figure 22:**
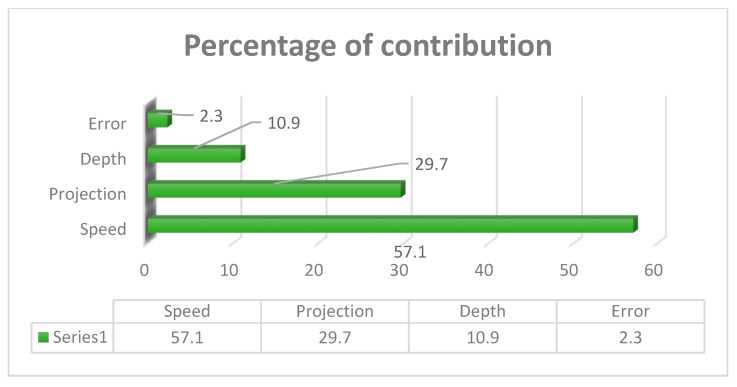
Percentage of contribution with-hole on the tube in the absence of the backing block.

Analysis of variance test was statistically considered, and it investigates the inheritance percentage of process parameters which influence the maximum tensile strength of the FWTPET process. In this investigation, the obtained results of ANOVA were tabulated in Table 20. The percentage of contribution of the most significate process parameter was tool rotational speed (57.10%) followed by tube projection (29.7%) and depth of cut (10.9%). The graph plot for interaction between process parameters was plotted and represented in Figure 21. The graphical video of the inheritance percentage was shown in Figure 22.

#### 4.4.3. Genetic Algorithm (GA) Approach

GA works under the principle of natural genetics that evaluates a set of points, and the essential element of GA consists of a chromosome and fitness value.

**Figure 23:**
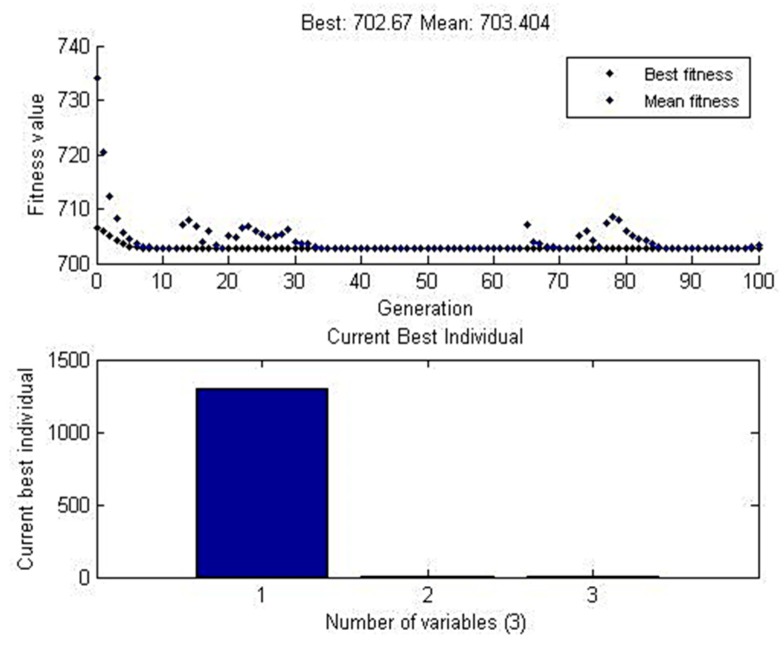
GA graph plot for data means with-hole on the tube in the absence of the backing block.

In the present research work, the GA predicts the value of maximum tensile strength as 702.67 MPa, and it adjusts to the data mean value 703.404 MPa shown in Figure 23. The input parameters were 1300 rpm speed, 1 mm projection and 0.5 mm depth of cut. The comparison table of tensile strength for predicted values and experimental values were tabulated in Table 21.

### 4.5. Comparison Statements

The comparison statements for all the workpieces WOHs and WHs on the circumference of the tube welded by employing backing block and in-absence of the backing block in the interference method. Firstly, to compare the tensile strength with experimental value and software predicted values and the comparison of the regression equation. Finally, to compare the percentage of contribution for all input parameters and hardness values. 

#### 4.5.1. Comparison of Tensile Strength

The comparison of tensile strength is made for with and without backing block arrangement welded by interference condition. Each method consists of two tube preparations. One was prepared with a hole in the circumference, and another one was tube without a hole on its circumference. Hence, the comparison statements was shown in Table 22. From the table, the software predicted value also concludes the maximum tensile strength was achieved by employing a backing block at the tube without a hole on its circumference. Also, the method was an interference fit method with heat dissipation more on the welding zone.

#### 4.5.2. Comparison of Regression Analysis

The maximum tensile strength was produced at 1300 rpm tool rotational speed, 1 mm tube projection, and 0.5 mm depth of cut for all research investigations. The accomplished value for this particular research work and its output values were calculated by the regression equation. Hence, equation one and equation two were provided in the particular research work under the condition, namely, with backing block, and equation three and equation four were provided under the condition of without backing block equation. By comparing both the condition, the tube WOH secures maximum tensile strength.

Tensile Strength = 393.9 + 0.3291 Speed − 17.4 Projection − 20.2 Depth(1)

Tensile Strength = 356.4 + 0.3247 Speed + 0.0 Projection − 36.3 Depth(2)

Tensile Strength = 431.1 + 0.2197 Speed + 4.5 Projection − 25.7 Depth(3)

Tensile Strength = 542.6 + 0.1292 Speed − 15.8 Projection − 26.3 Depth(4)

#### 4.5.3. Comparison of Percentage of Contribution

The percentage of contribution was calculated by statistical analysis of variance for the input parameters tool rotational speed, tube projection, and depth of cut. Hence, the comparison was done for two conditions one was employing backing block arrangement, and another one was in-absence of backing block arrangement—Table 23 shown the comparison statement of two conditions. By comparison, the tool rotational speed was contributed more to achieve maximum tensile strength.

#### 4.5.4. Comparison of Hardness Measurement

**Figure 24:**
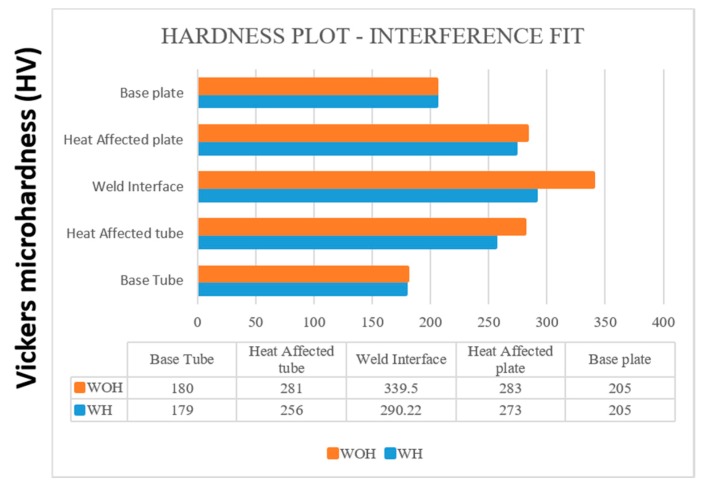
Hardness plot-Interference Fit for the tube with and WOH by employing backing block.

**Figure 25:**
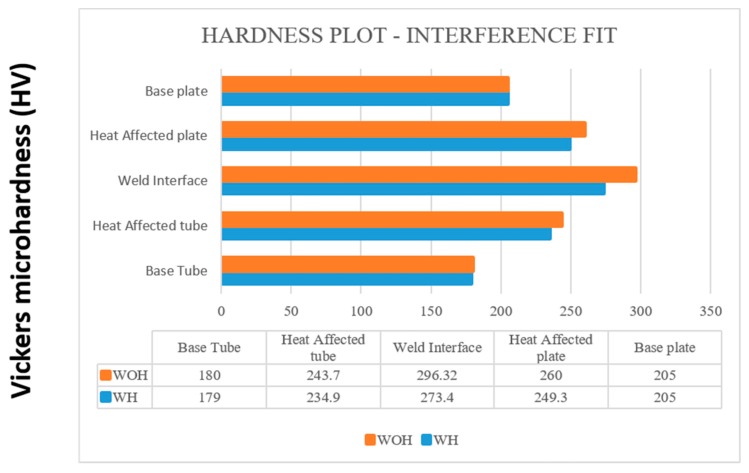
Hardness plot-Interference Fit for the tube with and without a hole in the absence of backing block.

The microhardness test was conducted by the Vicker hardness tester which has a load capacity of 1 kg, and the loading time was about 20 s for each sample. Figure 24 and Figure 25 show the comparison of hardness value for both with and without backing block arrangement. Each condition contains variations for tube preparations, which were compared by Table 24.

### 4.6. Special Characterization for Maximum Tensile Strength Welded Sample

#### 4.6.1. Optical Microscopic Examination

**Figure 26:**
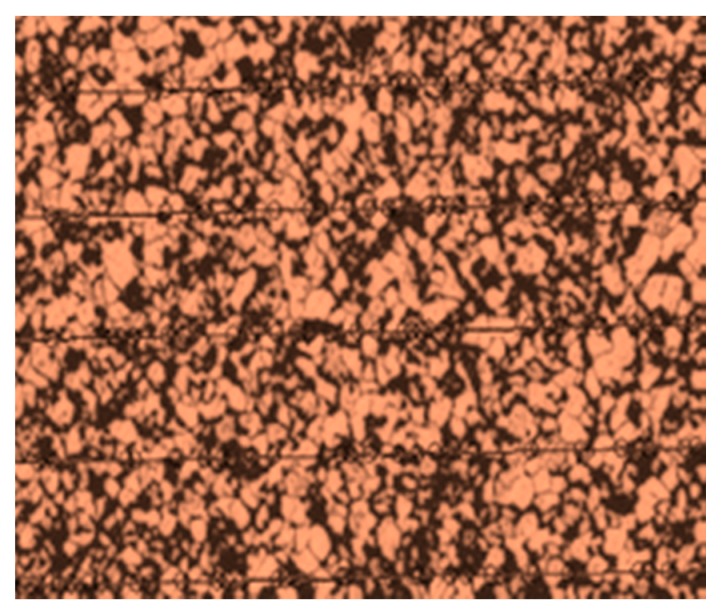
Grain size measurement of base metal—tube (36.9 µm).

**Figure 27:**
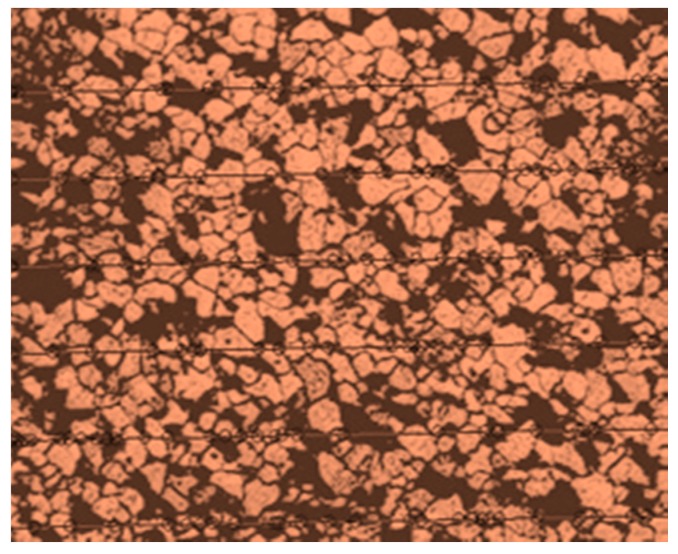
Grain size measurement of base metal—tube plate (52.3 µm).

**Figure 28:**
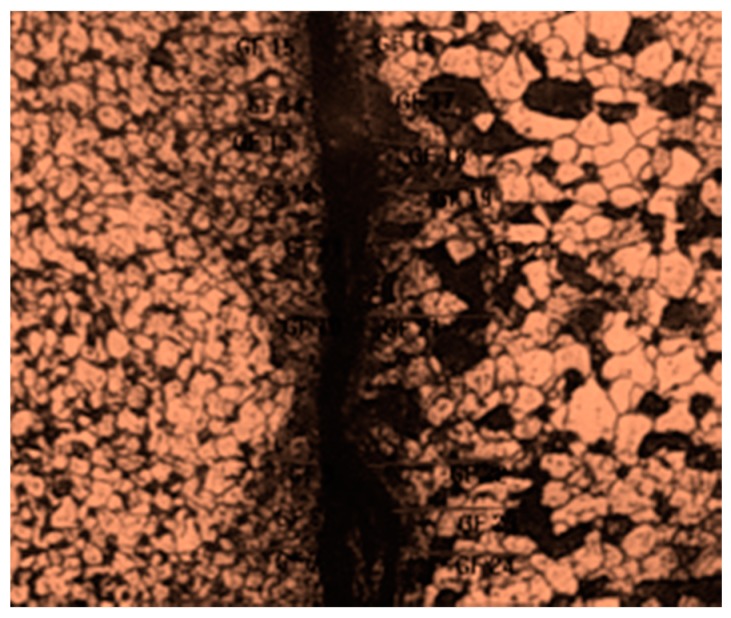
Grain size measurement of weld interface (19.919 µm).

The microstructure of the SA 213 tube to SA 387 tube plate has ferrite and pearlite. The Nital solution applied on the surface stimulates the structure. Figure 26 and Figure 27 were the base metal, and its grain size was measured as 36.9 µm and 52.3 µm in diameters. Figure 28 was the weld interface where the grains were coarsened because of heat dissipation through tube and tube plate. The grain size at the weld interface was 19.919 µm. These results indicate that the joint strength and hardness were more at the weld interface.

#### 4.6.2. Scanning Electron Microscope—Crack Analysis

The scanning electron microscope (Quanta 200, FEI Company, USA) was used to measure the bonding structure between the base metals. In this current work, the tool rotational speed was considered at 700 rpm, 1000 rpm, and 1300 rpm.

**Figure 29:**
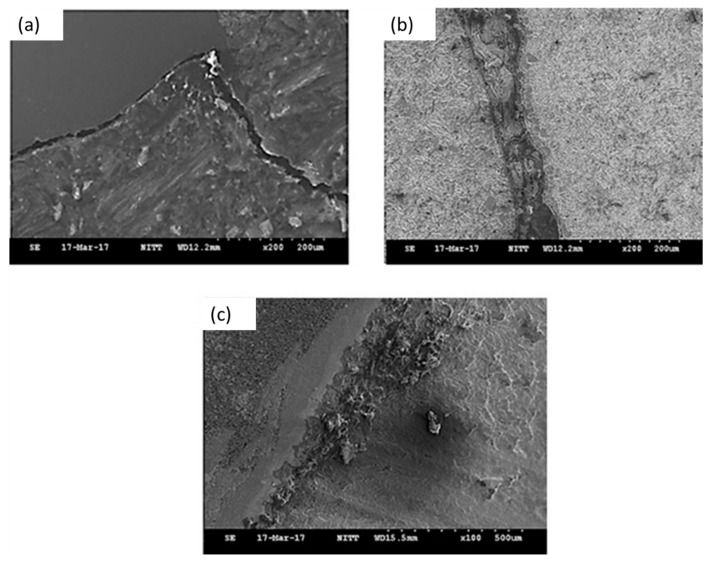
SEM images at various tool rotational speeds ((**a**) 700 rpm, (**b**) 1000 rpm, and (**c**) 1300 rpm).

At 700 rpm tool rotational speed, a large crack occurred at the interface is due to insufficient heat generated at low speed between the tube and the tube plate. At 1300 rpm, the optimum joint strength occurs due to sufficient heat generated by the FWTPET process. Figure 29 shows the scanning electron microscopic image for 700 rpm, 1000 rpm, and 1300 rpm tool rotational speeds. 

#### 4.6.3. Scanning Electron Microscope with EDS

**Figure 30:**
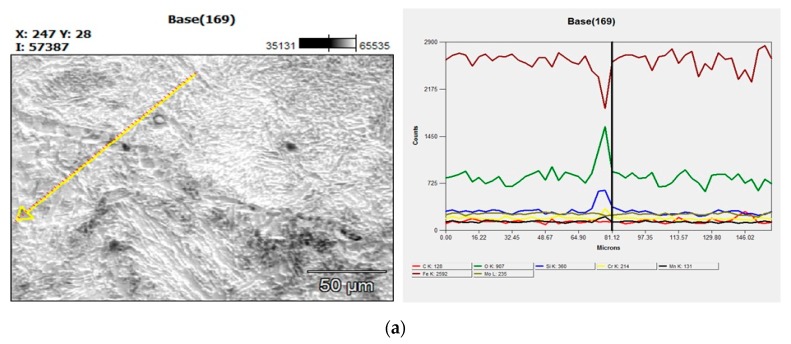

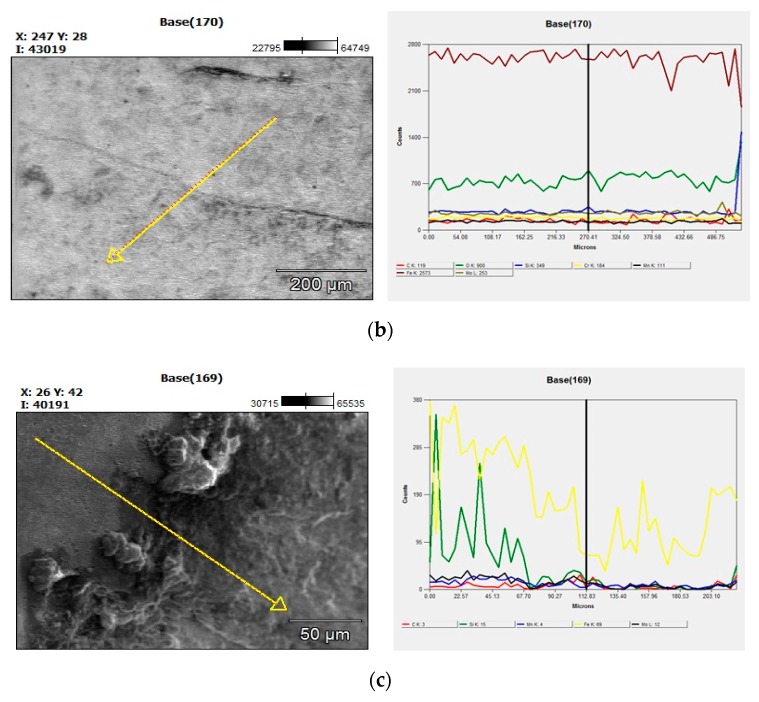
Scanning electron microscope with EDS at (**a**) 700 rpm, (**b**) 1000 rpm, (**c**) 1300 rpm

The EDS analysis (Quanta 200, FEI Company, Hillsboro, OR, USA) confirms the absence of intermetallic compounds at the weld interface obtained with different tool rotational speeds. Hence, increased hardness at the weld interface is due to the combined effect of grain refinement and severe plastic deformation between tube to tube plate. Figure 30 was shown the picture of the scanning electron microscope and EDS by varying tool rotational speed 700 rpm, 1000 rpm, and 1300 rpm.

#### 4.6.4. XRD Analysis

**Figure 31:**
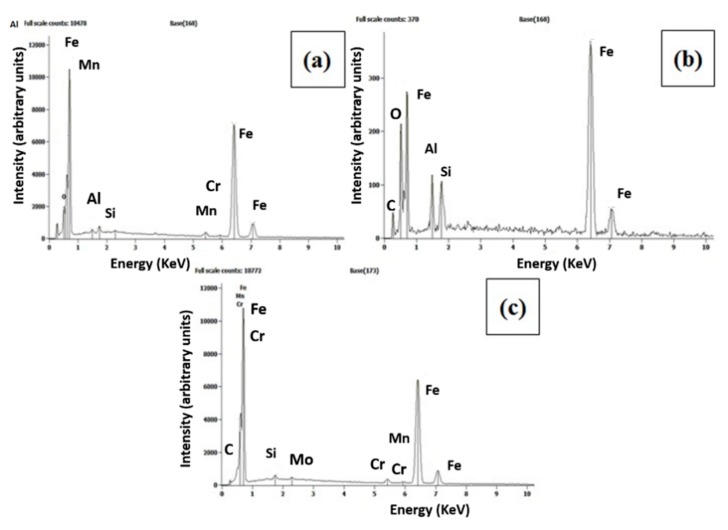
XRD analysis image at the weld interface (**a**) 700 rpm, (**b**) 1000 rpm, (**c**) 1300 rpm

The X-ray diffraction (A PANalytical^TM^ X’Pert PRO system, Malvern, UK) analysis was carried out to characterize the intermetallic compound at the weld interface. When the tool rotational speed was set at 1300 rpm, 1000 rpm, and 700 rpm, the XRD scan indicated the absence of intermetallic compounds. These are clearly reflected in Figure 31.

## 5. Conclusions

The joining of the SA213 tube to SA387 tube plate by the FWTPET technique was successfully carried out by the interference fit method with and without supporting block configuration. Further, the joining strength was evaluated and compared; further, optimization techniques such as Taguchi L_9_ and ANOVA were applied. The following are the main conclusions from this research study:

- Taguchi L_9_ orthogonal array technique suggested that the depth of cut was found to be a vital process parameter that determines the joining strength.
- It was observed that the optimum joining strength was found to be 836.8 MPa when the FWTPET technique was carried out with a backing block without a hole on the SA213 tube. The joining strength of 789.35 MPa was achieved when the same was carried out with a hole in the SA213 tube.
- Further, joining the strength of 700.8 MPa and 762.2 MPa was achieved without backing block arrangement with and without a hole on SA213 tube, respectively.
- The joining strength of 836.8 MPa was achieved, when the operating parameters of tool rotational speed, projection, and depth of cut were set at 1300 rpm, 1 mm, 0.5 mm respectively with the backing block, similarly the joining strength of 789.35 MPa was achieved when the operating parameters of tool rotational speed, projection, and depth of cut were set at 1300 rpm, 1 mm, 0.5 mm respectively without backing block.
- The result of ANOVA suggested that the percentage contribution of input parameters was tool rotational speed (73.08%), followed by the depth of cut (16.80%) and tube projection (7.57%). This result was achieved when the backing block was employed. Similarly, the ANOVA was applied for contribution in the absence of backing block was tool rotational speed (71.38%) followed by the depth of cut (22.6%) and tube projection (5.31%)
- The optimization techniques using GA was applied. Further, the experimental results were matched with the GA technique.

## Figures and Tables

**Table 1:** Alloy chemistry of SA213 tube and mechanical properties used in this study.

E	Mn	Cr	Mo	Ni	Cop	Ti	Fe	Mechanical Properties UTS (MPa)	%EI
Weight percentage	0.51	1.46	0.42	0.046	0.044	0.090	97.1	420	30

**Table 2:** Alloy chemistry of SA387 tube plate.

E	Mn	Cr	Mo	Va	Ti	Fe	Mechanical Properties UTS (MPa)	%El
Weight percentage	0.75	1.55	0.38	0.041	0.087	96.7	690	18

**Table 3:** Alloy chemistry of tungsten carbide.

E	C	H	Fe	O	Ni	Mo	Ti	V	Pd	W
wt%	0.08	0.015	0.25	0.13	0.75	4.31	0.4	4.5	0.2	89.365

**Table 4:** L_9_ orthogonal array factors and levels.

FACTOR	LEVELS		
	1	2	3
Speed (rpm)	700	1000	1300
Projection (mm)	0	1	2
Depth of Cut (mm)	0.2	0.4	0.6

**Table 5:** Design of experiments of L_9_ orthogonal array.

Experimental Number	Input Parameters		
	Tool Rotational Speed (rpm)	Tube Projection (mm)	Depth of Cut (mm)
1	700	0	0.5
2	700	1	1
3	700	2	1.5
4	1000	0	1
5	1000	1	1.5
6	1000	2	0.5
7	1300	0	1.5
8	1300	1	0.5
9	1300	2	1

**Table 6:** Input and output parameters of (WOH) on the tube using backing block.

Experiment Number	Input Parameters			Output Parameters
	Speed (rpm)	Projection (mm)	Depth (mm)	Tensile Strength (MPa)
1	700	0	0.5	612.35
2	700	1	1	580.35
3	700	2	1.5	565.65
4	1000	0	1	548.35
5	1000	1	1.5	711.45
6	1000	2	0.5	679.45
7	1300	0	1.5	791
8	1300	1	0.5	836.8
9	1300	2	1	723

**Table 7:** Response table for signal-to-noise (S/N) ratio of without-hole on the tube using backing block.

Level	Speed	Projection	Depth
1	55.35	56.16	56.95
2	56.16	56.92	55.75
3	57.87	56.29	56.69
Delta	2.51	0.76	1.20
Rank	1	3	2

**Table 8:** Analysis of variance of (WOH) on the tube using backing block.

Source	DF	Seq.SS	Adj.SS	Adj.MS	Percentage of Contribution
Speed rpm	2	61,455	61,455	30,728	73.08
Projection, mm	2	6369	6369	3185	7.57
Depth mm	2	14,129	14,129	7065	16.80
Error	2	2137	2137	1068	2.5
Total	8	84,090			100

**Table 9:** Comparison of experimental and software predicted value (WOH) in the tube using backing block.

Method	Input Parameters			Output Parameters
	Speed (rpm)	Projection (mm)	Depth (mm)	Tensile Strength (MPa)
GA	1270.666	0.5	1	837.4
Experimental	1300	0.5	1	836.8

**Table 10:** Input and output parameters with hole (WH) on the tube using backing block.

Experiment Number	Input Constraints			Output Constraints
	Speed (rpm)	Projection (mm)	Depth (mm)	Tensile Strength (MPa)
1	700	0	0.5	590.45
2	700	1	1	550.35
3	700	2	1.5	520.7
4	1000	0	1	596.35
5	1000	1	1.5	670.3
6	1000	2	0.5	630.15
7	1300	0	1.5	710.2
8	1300	1	0.5	789.35
9	1300	2	1	746.4

**Table 11:** Response table for Signal to Noise Ratios WH on the tube using backing block.

Level	Speed	Projection	Depth
1	54.86	55.99	56.45
2	56.01	56.43	55.93
3	57.48	55.93	55.96
Delta	2.62	0.50	0.53
Rank	1	3	2

**Table 12:** Results obtained from ANOVA of (WH) on the tube using backing block.

Source	DF	Seq.SS	Adj.SS	Adj.MS	Percentage of Contribution
Speed, rpm	2	57,650	57,650	28,825	87.3
Projection, mm	2	2831	2831	1416	4.30
Depth, mm	2	4838	4838	2419	7.40
Error	2	662	662	331	1.0
Total	8	65,982			100

**Table 13:** Comparison of experimental and software predicted value of (WH) on the tube using backing block.

Method	Input Parameters			Output Parameters
	Speed (rpm)	Projection (mm)	Depth (mm)	Tensile Strength (MPa)
Genetic Algorithm	1300	0.5	1	786.95
Experimental	1300	0.5	1	789.35

**Table 14:** Input and output parameters of (WOH) on the tube in-absence of the backing block.

Experiment Number	Input Parameters			Output Parameters
	Speed (rpm)	Projection (mm)	Depth (mm)	Tensile Strength (MPa)
1	700	0	0.5	582.5
2	700	1	1	525.7
3	700	2	1.5	578.9
4	1000	0	1	610.7
5	1000	1	1.5	661.1
6	1000	2	0.5	624.3
7	1300	0	1.5	651.8
8	1300	1	0.5	762.2
9	1300	2	1	668.6

**Table 15:** Response table for S/N ratio of without-hole on the tube in-absence of the backing block.

Level	Speed	Projection	Depth
1	54.99	55.77	56.29
2	56.01	56.15	55.54
3	56.81	55.89	55.98
Delta	1.82	0.39	0.74
Rank	1	3	2

**Table 16:** Analysis of variance of without-hole on the tube in-absence of the backing block.

Source	DF	Seq.SS	Adj.SS	Adj.MS	F	Percentage of Contribution
Speed, rpm	2	26,098	26,098	13,049.1	324,849.35	71.38
Projection, mm	2	1944	1944	971.9	337,748.35	5.32
Depth, mm	2	8288	8288	2243.9	1,703,186.23	22.6
Error	2	228	228	2014.2		0.7
Total	8	36,558				100

**Table 17:** Comparison of experimental and software predicted value (WOH) in the tube in the absence of the backing block.

Method	Input Parameters			Output Parameters
	Speed (rpm)	Projection (mm)	Depth (mm)	Tensile Strength (MPa)
GA	1300	0.5	1	771.3
EXPERIMENTAL	1300	0.5	1	762.2

**Table 18:** Input and output parameters of with-hole on the tube in-absence of the backing block.

Experiment Number	Input Constraints			Output Constraints
	Speed (rpm)	Projection (mm)	Depth (mm)	Tensile Strength (MPa)
1	700	0	0.5	610.3
2	700	1	1	597.63
3	700	2	1.5	536.4
4	1000	0	1	650.8
5	1000	1	1.5	680.3
6	1000	2	0.5	615
7	1300	0	1.5	630.5
8	1300	1	0.5	700.8
9	1300	2	1	645.6

**Table 19:** Response table for the signal to noise ratios with-hole on the tube in the absence of backing block.

Level	Speed	Projection	Depth
1	55.28	55.99	56.13
2	56.23	56.36	56.00
3	56.37	55.52	55.75
Delta	1.09	0.84	0.39
Rank	1	2	3

**Table 20:** Results obtained from ANOVA with-hole on the tube in the absence of the backing block.

Source	DF	Seq.SS	Adj.SS	Adj.MS	Percentage Of Contribution
Speed, rpm	2	10,639	10,639	5319.4	57.10
Projection, mm	2	5507	5507	2753.7	29.7
Depth, mm	2	2050	2050	1025	10.9
Error	2	444	444	222	2.3
Total	8	31,129			100

**Table 21:** Comparison of experimental and software predicted value with a hole in the tube in the absence of the backing block.

Method	Input Parameters			Output Parameters
	Speed (rpm)	Projection (mm)	Depth (mm)	Tensile Strength (MPa)
GA	1300	0.5	1	702.67
Experimental	1300	0.5	1	700.8

**Table 22:** Comparison between WOHs and WHs on the circumference of the tube using the backing block.

Conditions	Tube Preparations	Method of Analysis	Input Specifications			Output Specifications
			Speed mm	Projection mm	Depth mm	Tensile Strength MPa
Using backing block	WOH	GA	1300	1	0.5	837.4
		Experimental	1300	1	0.5	836.8
	WH	GA	1300	1	0.5	786.95
		Experimental	1300	1	0.5	789.35
Without using backing block	WOH	GA	1300	1	0.5	771.3
		Experimental	1300	1	0.5	762.2
	WH	GA	1300	1	0.5	702.67
		Experimental	1300	1	0.5	700.8

**Table 23:** Inheritance % of most influencing factors using backing block.

Parameters	Percentage of Inheritance (%)			
	Employing Backing Block		In the Absence of Backing Block	
Tube Preparation	WOHs	WHs	WOHs	WHs
Speed (rpm)	73.08	87.3	71.38	57.10
Projection (mm)	7.57	4.30	5.32	29.7
Depth (mm)	16.80	7.40	22.6	10.9

**Table 24:** Hardness tests comparison table (HV).

Conditions	Tube Preparation	Base Tube	Heat Affected Tube	Weld Interface	Heat Affected Plate	Baseplate
With backing block	WH	179	256	290.22	273	205
	WOH	180	281	339.50	283	205
Without backing block	WH	179	234.90	273.40	249.3	205
	WOH	180	243.7	296.32	260	205
